# The Experimental Treatment of Corneal Graft Rejection with the Interleukin-1 Receptor Antagonist (IL-1ra) Gene

**DOI:** 10.1371/journal.pone.0060714

**Published:** 2013-05-28

**Authors:** Jin Yuan, Yi Liu, Weilan Huang, Shiyou Zhou, Shiqi Ling, Jiaqi Chen

**Affiliations:** 1 State Key Laboratory of Ophthalmology, Zhongshan Ophthalmic Centre, SunYat-sen University, Guangzhou, Guangdong Province, China; 2 Department of Ophthalmology, Nanjing Hospital of Traditional Chinese Medicine, Nanjing, Jiangsu Province, China; 3 The Third Affiliated Hospital, Sun Yat-sen University, Guangzhou, Guangdong Province, China; University of Florida, United States of America

## Abstract

**Purpose:**

To investigate the protective effects of interleukin-1 receptor antagonist (IL-1ra) gene transfer in a rat model of corneal graft rejection.

**Methods:**

We constructed a recombinant plasmid (pcDNA3.1-hIL-1ra) with high IL-1ra expression in eukaryotic cells. Using a Wistar-SD rat model of corneal graft rejection, we examined the effects of IL-1ra in vivo after cationic polymer jetPEI-mediated nonviral gene delivery. Four groups were included: negative controls (group I, n = 20), pcDNA3.1-hIL-1ra corneal stromal injection (group II, n = 34), pcDNA3.1-hIL-1ra anterior chamber injection (group III, n = 34), and 500 µg/ml IL-1ra protein subconjunctiva injection (group IV, n = 20). IL-1ra expression after transfection was evaluated by real-time polymerase chain reaction (RT-PCR) and western blotting. The rejection indices of corneal grafts were analysed in the different groups. The expression levels of transforming growth factor β1 (TGF-β1), inflammatory chemokines including RANTES, interleukin-1 (IL-1) and the numbers of CD4+ and CD8+ T cells in the grafts were determined by biochemical assays at different time points after corneal transplantation.

**Results:**

Various degrees of inflammatory cell infiltration and graft neovascularisation were observed by histopathology. After injecting the pcDNA3.1-hIL-1ra plasmid into the cornea, IL-1ra mRNA and protein expression was detected in the corneal stroma and reached a peak on day 3. The graft survival curves indicated that the corneal transparency rates of grafts in the IL-1ra gene-treated group and the IL-1ra protein-treated group were higher compared with the untreated group (P<0.05). During the period of acute rejection, TGF-β1, RANTES, IL-1α and IL-1β levels in the grafts in the IL-1ra treatment groups were lower than the control group (P<0.05). CD4+ and CD8+ T cell counts were reduced significantly in the corneal grafts of groups II, III and IV compared with group I (P<0.05).

**Conclusion:**

Interleukin-1 receptor antagonist (IL-1ra) gene transfer treatment inhibits graft rejection after corneal transplantation through the downregulation of immune mediators.

## Introduction

The cornea is avascular and, as such, is an “immune privileged” site. Therefore, the corneal transplant success rate is the highest among organ transplants [1]–[2]. However, immune responses are the primary cause for graft failure in penetrating keratoplasty [3]. Interleukin-1 (IL-1) is the key cytokine participating in corneal transplant rejection [4]. In keratoplasty, increasing the IL-1 level causes antigen-presenting cell (e.g., Langerhans cells and lymphocytes) aggregation and neutrophil infiltration, leading to inflammation. Thus, antagonising the biological activity of IL-1 could effectively prolong graft survival. Interleukin-1 receptor antagonist (IL-1ra) is a natural inhibitor of IL-1. Recent studies have indicated [5]–[8] that IL-1ra may be effective for treating the rejection of corneal transplants. Jie [9] demonstrated that the subconjunctival injection of large doses of IL-1ra could prolong the survival of rat allografts following high-risk corneal transplantation. Although IL-1ra clearly inhibits immune and inflammatory reactions, as a natural protein, the cytokine is not stable for clinical applications, and its subconjunctival injection may increase patient suffering.

In a previous study, we used genetic engineering techniques to construct a recombinant plasmid with the human IL-1ra cDNA sequence that could effectively express high levels of IL-1ra mRNA and protein in eukaryotic cells. The IL-1ra plasmid DNA was then delivered using a new type of non-viral cationic polymer vector into the corneal stroma and anterior chamber. We observed exogenous gene expression in rat eye tissues and assessed the effectiveness and safety of in situ corneal transfection of the exogenous gene [10]. We hope that our findings will provide a novel transgenic therapy for corneal donor rejection by inducing high IL-1ra expression in the graft.

## Materials and Methods

### Reagents/Equipment

The following reagents were purchased from the indicated companies: cationic polymer jetPEI in vivo (Poly-plus Transfection, France); rat IL-1α and IL-1β ELISA kits (Bioscience, USA); TRIzol extraction reagent (Invitrogen, USA); total RNA extraction kit (TakaRa, USA); mouse anti-human IL-1ra antibody (Cytoskeleton, USA); biological microscope (Olympus BH-2, Japan); surgical microscope (Topcon-300, Japan); slit-lamp microscope (Topcon, Japan); ultra-microtome (LKB-V, Sweden); gel imaging system (BioRad, USA); and PCR instrument (PE, USA).

### Experimental animals

Research animals were obtained and cared for in accordance with the recommendations of the Guide for the Care and Use of Laboratory Animals, Institute of Laboratory Animal Resources, and the Public Health Service Policy on the Humane Care and Use of Laboratory Animals.

Allograft corneal transplantation was performed between host Wistar (SD) (n = 108) and Sprague Dawley donor rats (n = 54). Rats were provided by the Experimental Animal Centre of Guangdong Province. Pathological changes in the optical adnexal and anterior segment of both rat eyes were excluded under slit-lamp microscopy. Tobramycin (0.3%) eye drops (Tobrex, Alcon, Belgium) were administered daily to the eyes of all animals (3 times/day) for three days to prevent infection after surgery.

The Wistar-SD rat model of corneal graft rejection [11]: The rat pupil was dilated with the compound tropicamide(0.5%) eye drops (Tropicamide Phenylephrine Eye Drops, Santen, Japan) 20 minutes before surgery, and the animal was anaesthetised with an intraperitoneal injection of 0.2 mL/100 g ammonia ketamine + chlorpromazine (1:1). The corneal surface was anaesthetised with proparacaine(0.5%) eye drops (Alcon, Belgium). Sterile surgery was performed under a surgical microscope. The corneal graft was obtained from the donor SD rat with 3.25-mm trephine. The graft was placed in a sterile Petri dish, and sodium hyaluronate was added to protect the endothelial surface. The upper and lower eyelids of the Wistar rats were retracted, and with a 3.0-mm diameter trephine, the centre of the cornea was cut, and the graft was placed on the host bed. The wound was sutured with 10–0 nylon stitches using an interrupted suture technique, the anterior chamber was reconstructed by BSS, and the knot was exposed. After surgery, the pupil was dilated, 2000 U of gentamicin was injected subconjunctivally, and the palpebral margins were sutured. The eyelid suture was removed after 24 hours for drug administration.

### Animal grouping and gene transfer protocol

The PEI/DNA transfection mixture was formulated as 20 µg of plasmid in 10 µL of mixture, and the mixture was incubated at room temperature for 30 minutes before use. The concentration of hIL-1ra purified protein solution was 1.5 mg/mL, which was diluted to 500 µg/mL with normal saline and stored at 4°C.

The animals were divided into 4 groups. Group I (n = 20) was the negative control, which received a subconjunctival injection of saline after surgery. Group II (n = 34) was the IL-1ra gene corneal injection group, which received a 20 µg injection of PEI/DNA mixture into the corneal stroma before donor graft collection [10]. Group III (n = 34) was the IL-1ra gene anterior chamber injection group, which received an injection of 20 µg of the PEI/DNA mixture into the anterior chamber after graft-graft bed alignment suturing. Group IV (n = 20) was the positive control, which received a subconjunctival injection of IL-1ra protein after surgery. We excluded animals with infection, hyphaema, anterior chamber disappearance caused by surgery or severe corneal graft oedema, and/or opacity and disruption within 5 days after surgery. Several animals died from overdoses of anaesthetic or apparent graft infection, and these experimental animals were replaced over time.

Before immune rejection of the corneal grafts (i.e., 6 days after surgery), 2 animals in groups I and IV and 4 in groups II and III were sacrificed. During immune rejection of the corneal grafts, 8 animals in groups I and IV and 12 in groups II and III were sacrificed. The remaining animals were sacrificed 2 weeks after graft rejection.

### Corneal graft observation and evaluation after surgery

Based on the scoring criteria of Larkin [12], the corneal grafts were scored using three indices: opacity, oedema and neovascularisation. The sum of the scores for these 3 indicators was the rejection index (RI). An RI ≥5 or a corneal oedema score of 3 was defined as the occurrence of immune rejection.

### Graft histopathology

Corneal grafts were placed in 10% formalin, dehydrated with routine methods and embedded in paraffin. Samples were serially sectioned into 5-µm-thick slices. After haematoxylin-eosin staining, the sections were mounted with neutral balsam. The pathological changes in each layer of the cornea were observed under optical microscopy.

### Immunohistochemistry

After deparaffinisation for 30 min, the samples were cleared using a graded ethanol series of 95%, 90% and 85%. The specimen was then placed in sodium citrate solution and subjected to microwave antigen retrieval for 10 min, followed by three 5-min irrigations in PBS. Specimens were examined by immunohistochemistry in accordance with the instructions of the SP reagent kits. Antibodies against transforming growth factor β1 (TGF-β1), RANTES and CD4/CD8 T cells were used as the primary antibodies (1:100 dilution) and were allowed to bind for 5 min, followed by irrigation with distilled water. The specimen was then mounted onto a slide using neutral balsam and observed under the microscope. The negative control sample was prepared in the same manner, except that PBS was used in place of the primary antibody solution. The results were interpreted as follows: clear cell membrane boundary with no specific staining (−); light brown, mild specific staining in the cell membrane or cytoplasm (+); brown, moderate specific staining in the cell membrane or cytoplasm (++); significant, specific staining in the cell membrane or cytoplasm with brown or dark brown colouring (+++). The average number of positive T cells was counted in the central areas of the corneal grafts at low magnification.

### Detection of IL-1α and IL-1β in corneal grafts

Samples were ground in liquid nitrogen, and the tissue debris of each cornea was resuspended in 1 mL PBS and centrifuged at 1500 gram at 4°C for 10 minutes. The supernatant was collected into EP tubes and preserved at −80°C.

Tissue samples and frozen rat recombinant IL-1α and IL-1β standards from E. coli were added to a 96-well plate coated with anti-rat IL-1α and IL-1β antibodies. Staining was performed as previously described. The results were read by a microplate reader (BIO-RAD) at 450 nm for analysis.

### Detection of IL-1ra protein and mRNA in corneal grafts

The corneal grafts from groups II and III were collected and tested by Western blotting and PCR. The procedures for corneal total protein extraction and the detection of IL-1ra protein were performed as previously described [10]. Purified IL-1ra protein was used as a positive control, and pcDNA3.1 empty vector was used as a negative control for electrophoresis. A predetermined amount of corneal RNA was used in each reaction. The PCR product was used for electrophoresis and run four times. The concentration of gel-extracted DNA was then determined by measuring the OD.

### Statistical analysis

All data were expressed as the mean ± standard deviation. A one-way ANOVA was used for multiple comparisons within the same group. A t-test was used for differences between two groups. P<0.05 was considered to indicate statistical significance.

## Results

### Corneal graft survival time

**Figure 1:**
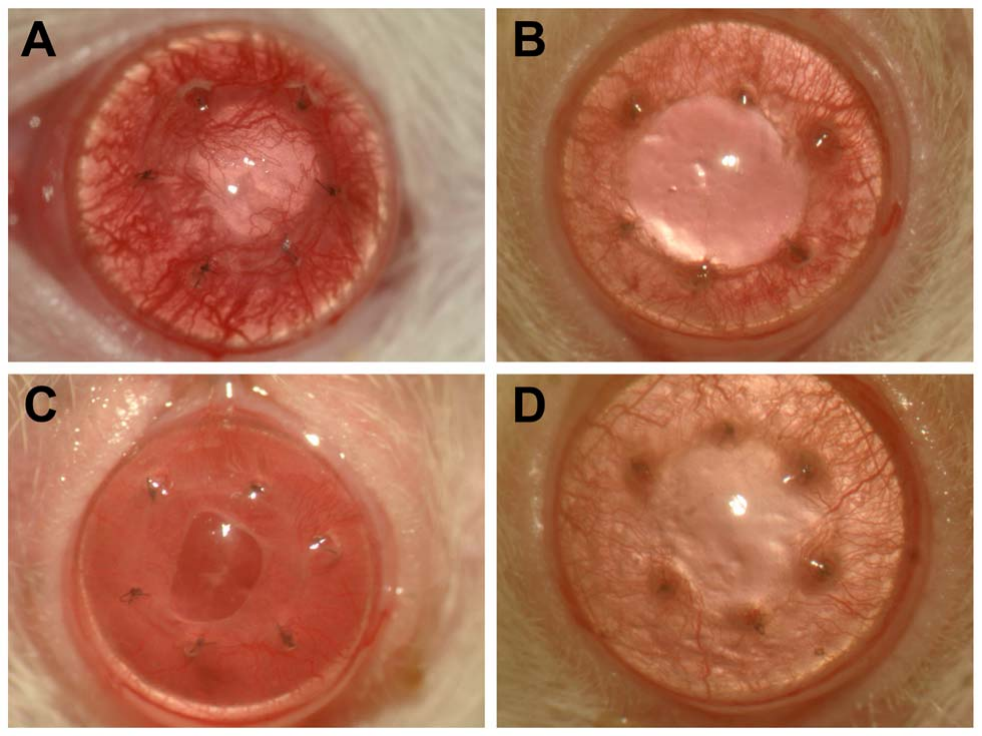
The appearance of the corneal graft 14 days after the operation. A, In group I, the graft showed oedema and new blood vessel growth into the centre of the graft. B, in the group II, the graft showed mild oedema, and fewer new blood vessels were observed than in controls. C-D, in the groups III and group IV, the graft was transparent, and no neovascularisation was found in the centre of the graft.

At 3 days post-surgery, new vessels began to appear in the graft bed. The wound interface between the graft and graft bed showed mild oedema, and the graft was transparent. At days 6–7 post-surgery, the junction between the graft and graft bed showed neovascularisation in all the rats. In the control group, newly grown vessels entered the graft centre on the ninth day. The duration of rejection was inconsistent, but in general, the corneal oedema and opacity began to diminish after 1–2 weeks, and the neovascularisation began to subside (Fig. 1). Analysis of variance (ANOVA) showed that the survival times of the grafts in groups II, III, and IV were significantly longer than those of the grafts in group I (P<0.05), and there was no significant difference within each treatment group(table 1).

**Table 1:** The onset time of graft rejection (days).

Group I	Group II	Group III	Group IV	F	P
7.65±0.88	12.56±1.31	12.21±1.14	11.69±2.37	77.984	0.000

In days; mean ± standard deviation P<0.05.

(P = 0.824, Table 2).

**Figure 2:**
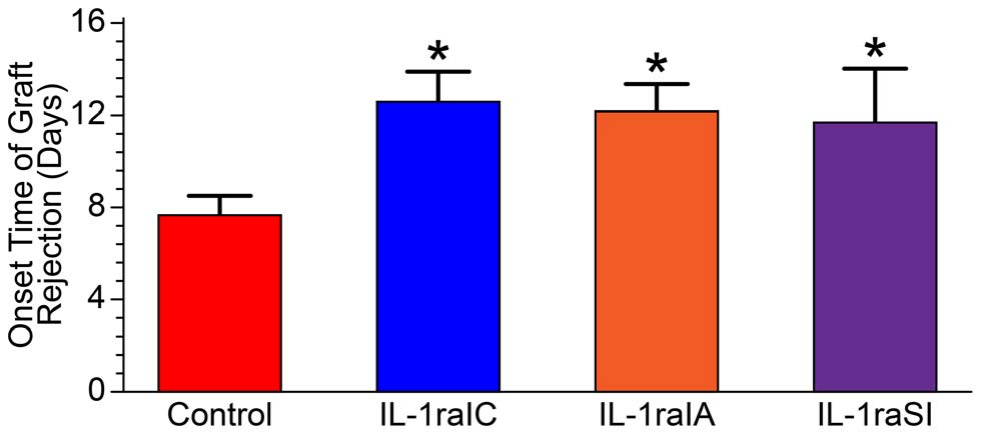
The survival curve of the grafts for the four groups. The recipients in group I exhibited accelerated rejection. The median survival was significantly different among the four groups according to a log-rank test (p<0.01).

The graft survival curves suggested that the corneal transparency rates of grafts in the IL-1ra gene-treated group and the IL-1ra protein-treated group were better and higher than that of the untreated group (P<0.05). The rate of rejection in the IL-1ra gene-treated group was less than that of the IL-1ra protein-treated group 12 days after the operation (Fig. 2).

Two weeks after surgery, the scores for opacity, stromal oedema, and neovascularisation in the experimental groups were lower than those of the control group (P<0.05), and the rejection indices were significantly different (P<0.05). The differences in graft rejection indices within the treatment groups were not significant (P = 0.824, Table 2).

**Table 2:** Scores on corneal transplant indices 14 days after surgery.*

Group	Transparency	Stromal Edema	Neovascularization	Rejection Index
Group I	2.88±0.64	1.88±0.35	3.00±0.54	7.75±0.45
Group II	2.00±0.43	1.25±0.45	2.00±0.95	5.25±1.14
Group III	2.08±0.29	1.33±0.49	2.08±0.52	5.50±1.00
Group IV	2.00±0.54	1.25±0.46	2.00±0.54	5.38±0.74
F	7.097	3.799	4.298	14.292
P	0.001	0.018	0.011	0.000

* Mean ± standard deviation.

F = Fisher T-test values.

P = probability value.

### Graft histopathology

**Figure 3:**
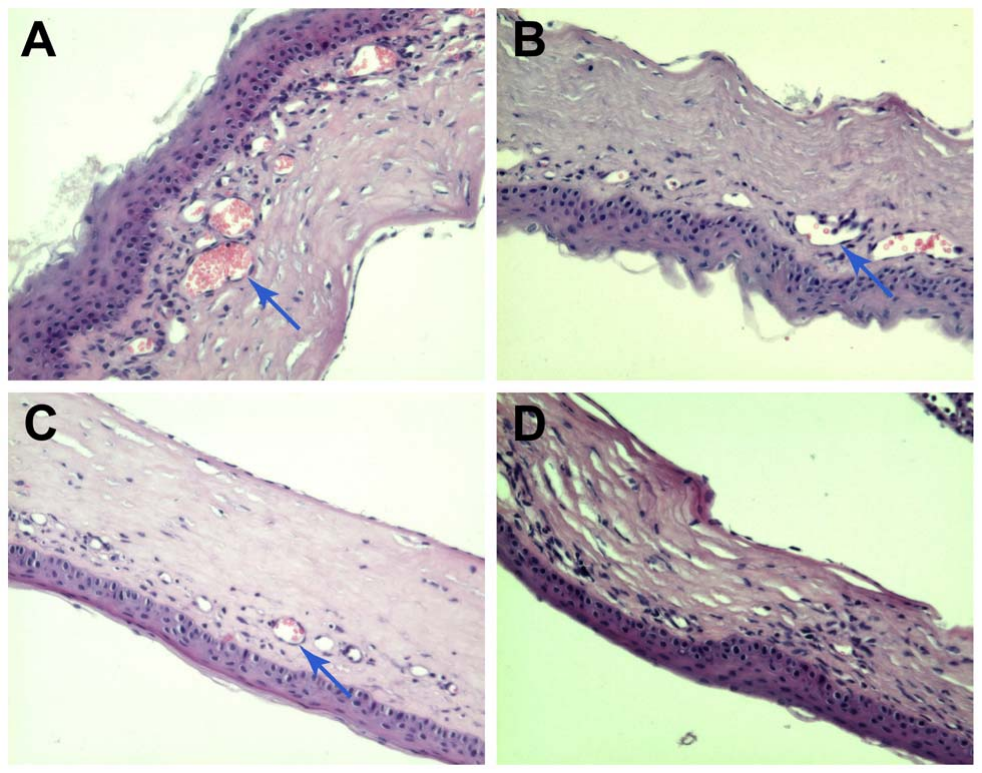
HE staining at 2 weeks after corneal transplantation. A, Obvious graft oedema, inflammatory cell infiltration and neovascularisation (blue arrow) in the stroma were observed in the group I. B-D(group II∼group IV), Mild oedema and reduced lymphocyte infiltration and neovascularisation were observed in the IL-1ra gene treatment groups. (×200).

During the period of acute graft rejection, obvious graft oedema, lymphocyte infiltration and neovascularisation in the corneal stroma were observed in the controls, whereas mild oedema and reduced lymphocyte infiltration and neovascularisation were observed in the IL-1ra gene treatment groups. Two weeks after rejection, oedema and the degree of inflammatory cell infiltration were also reduced. Compared with the treatment groups, the degree of rejection remained more prominent in the control groups, as judged by the degree of neovascularisation and inflammatory cell infiltration (Figure 3).

### Graft expression of TGF-β1

**Figure 4:**
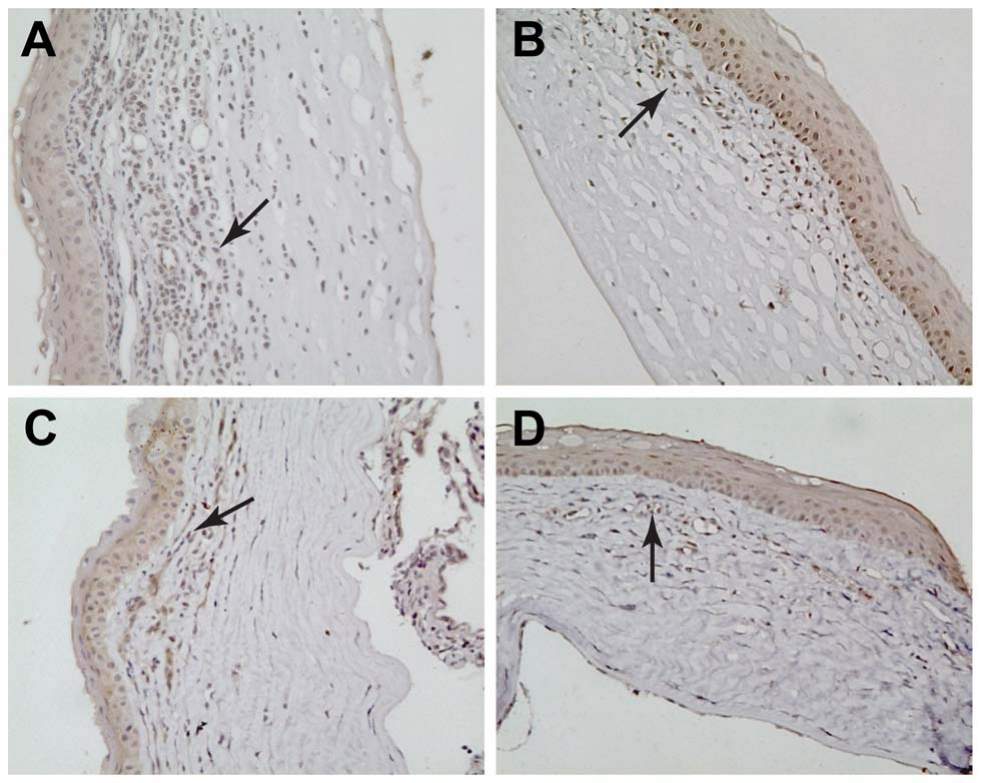
TGF-β1 expression in the corneal grafts. TGF-β1 is shown by dark brown staining (dark arrows) in the cytoplasm. B–D,(group II∼group IV), the quantity of positive inflammatory cells was lower than that of the grafts in the A (group I). (×200).

During the acute corneal rejection, there was extensive TGF-β1 expression in the corneal grafts from rats in the negative control group. In addition, TGF-β1 was also expressed in the corneal stroma, endothelial cells, and some inflammatory cells, which showed dark brown staining (+++). Specifically, in the corneal grafts of groups II, III, and IV, the basal layer of corneal epithelial cells and fibroblasts and the cytoplasm of corneal endothelial cells showed light yellowish-brown staining (+). The quantity of positive inflammatory cells was lower than that of the rats in the control group (Fig 4).

### Graft expression of RANTES

**Figure 5:**
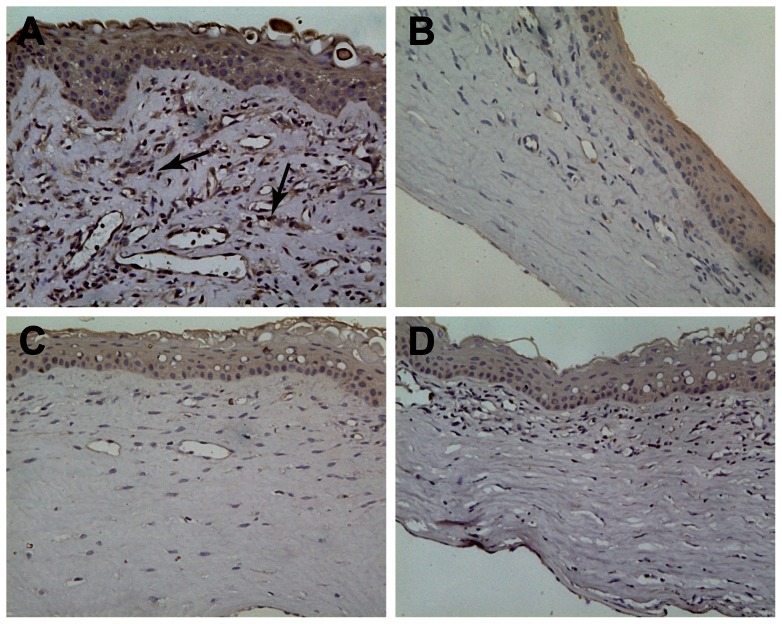
RANTES expression in the corneal grafts. RANTES was expressed in the cell membrane and cytoplasm and is shown by dark brown staining (dark arrows). The expression intensities of RAβ1NTES in the group I(A) were increased compared to groups II, III and IV(B-D). (×200).

During acute corneal rejection, RANTES expression was observed in the cell membrane and cytoplasm. The average colour intensities of the corneal epithelium, neovascular basement membrane and the few inflammatory cells in the control group were increased compared to groups II, III and IV (Fig 5).

### CD4 and CD8 T cell graft infiltration

**Figure 6:**
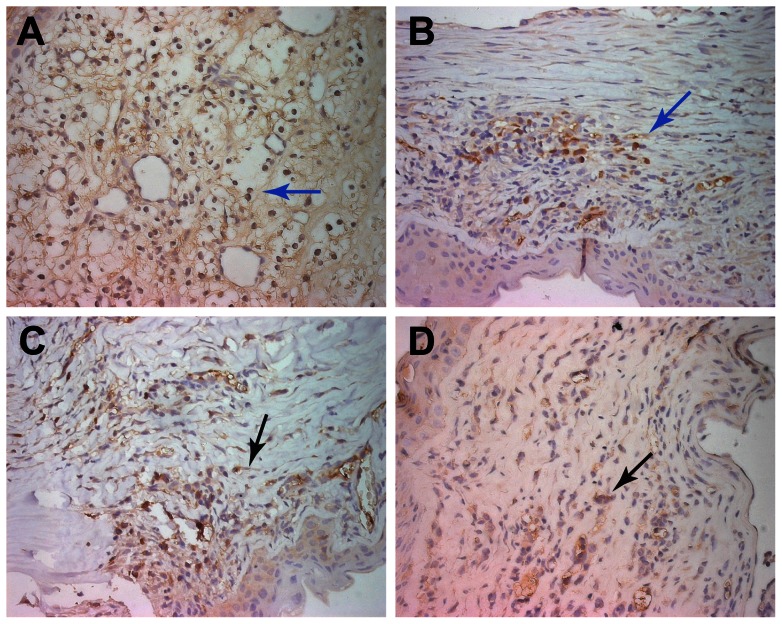
The expression of CD4+cells in the corneal graft. During acute corneal rejection, increased CD4+ cells were observed in all of the groups compared to pre-operation. Brownish-yellow staining in the cytoplasm can be observed(black and blue arrow). The numbers of CD4+ cells in the group I (A)were higher than other groups. There was no significant difference between treatment groups(B-D) (×400).

Before acute corneal rejection, there were only a few CD4+ cells in the control group. During acute corneal rejection, there were many CD4+ and CD8+ cells in all of the groups. Furthermore, the numbers of CD4+ and CD8+ cells in the control group were higher than those in groups II, III and IV. There was no significant difference in the experimental groups (Figures 6, Table 3).

**Table 3:** CD4^+^ and CD8^+^ T cell counts in graft.

	Before Acute Rejection		Acute Rejection		Two Weeks After Acute Rejection	
	CD4^+^ cell count*	CD8^+^ cell count*	CD4^+^ cell count*	CD8^+^ cell count*	CD4^+^ cell count*	CD8^+^ cell count*
Group I	14.87±0.31	10.13±0.16	20.53±3.24	15.50±0.68	24.75±0.50	15.8±0.37
Group II	9.40±0.43	5.93±0.46	11.60±0.91	9.25±0.62	15.15±0.34	8.10±0.81
Group III	7.50±0.93	6.25±0.30	11.45±1.31	9.40±0.63	15.65±0.38	7.25±0.19
Group IV	7.93±0.99	5.93±0.50	11.20±1.50	9.30±0.60	9.75±0.84	4.70±0.48
F	68.743	92.377	21.795	95.029	510.099	348.299
P	<0.001	<0.001	<0.001	<0.001	<0.001	<0.001

* Mean ± standard deviation.

### Detection of IL-1α and IL-1β in corneal grafts

**Figure 7:**
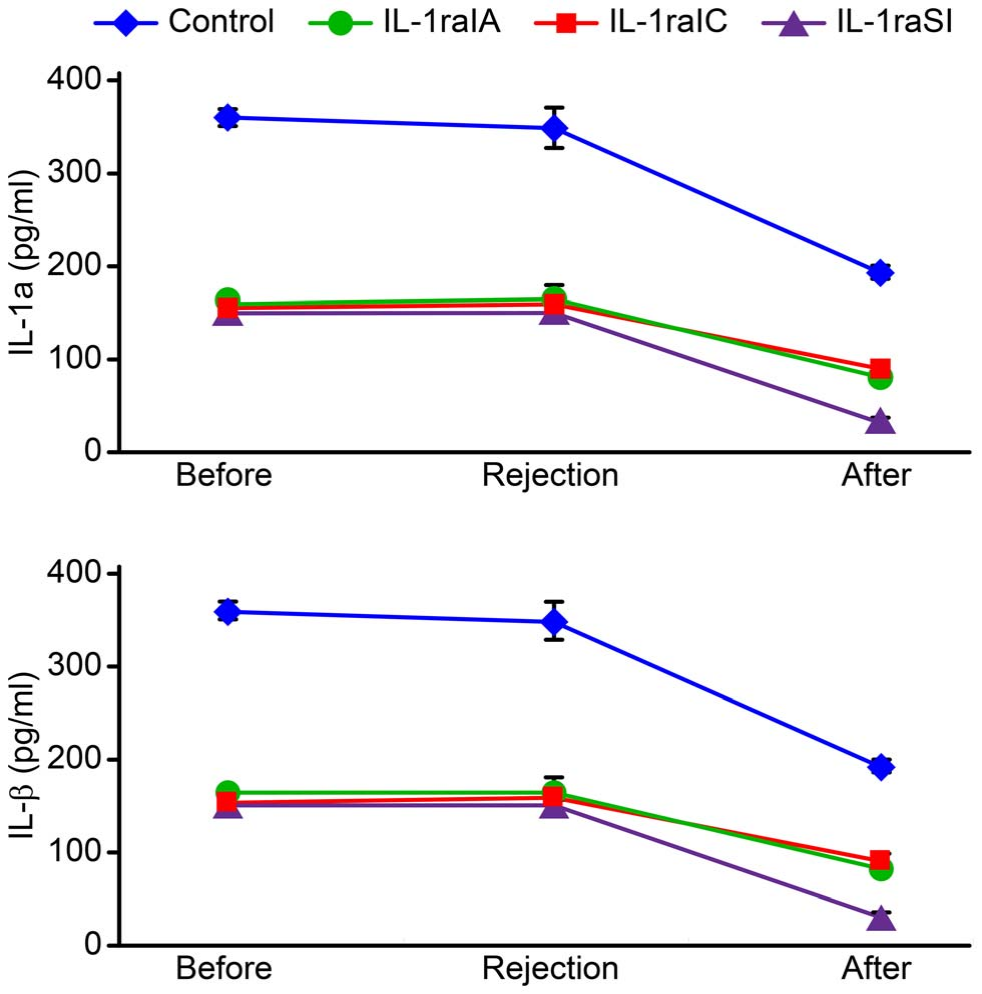
The concentrations of IL-1α and IL-1β in the cornea. The levels of IL-1α and IL-1β in the graft peaked before rejection and gradually decreased after rejection. Two weeks after rejection, the concentrations of IL-1α and IL-1β remained high in group I. The concentrations of IL-1α and IL-1β in the IL-1ra treatment groups(II∼III) at all time points were significantly lower than those in the group I.

During acute corneal rejection, IL-1α and IL-1β expression in the IL-1ra gene treatment groups was lower than that in the negative control group (P<0.05), and there was no significant difference between the IL-1ra treatment groups (P = 0.292 and 0.394). Two weeks after rejection, the IL-1α and IL-1β levels in groups II, III, and IV were lower than those in group I (P<0.05). The IL-1α and IL-1β levels in groups II and III were significantly different from those in group IV; however, there was no significant difference between groups II and III (P = 0.066, 0.166) (Fig. 7).

### Detection of IL-1ra protein and mRNA in corneal grafts

**Figure 8:**
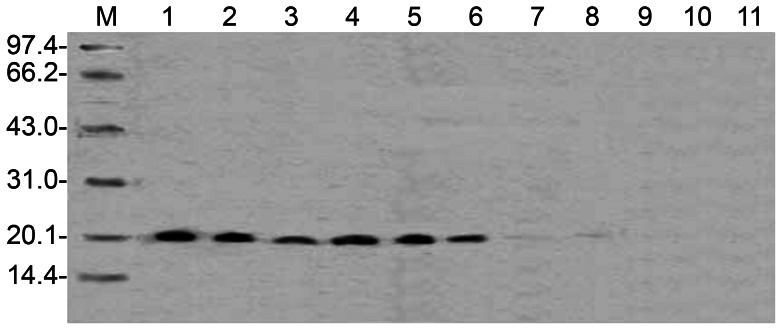
IL-1ra protein expression in the corneal grafts transferred by the IL-1ra gene. **IL-1ra protein maintained a higher level of expression in the grafts before and during acute rejection and decreased after acute rejection.** Lanes 1, 2, 3, and 7: anterior chamber injection(group III); lanes 4, 5, 6, and 8: intrastromal injection(group II); from left to right: 3 days, before acute rejection, acute rejection, and two weeks after acute rejection.

Corneal grafts injected with the IL-1ra gene in the anterior chamber (group III) showed IL-1ra protein expression at post-operative day 3. After acute rejection, IL-1ra protein expression was weak in the corneas of the group that underwent anterior chamber injection; IL-1ra expression was also low in the group that received a PEI/DNA injection in the corneal stroma 1 hour before donor graft collection (group II) (Fig 8).

**Figure 9:**
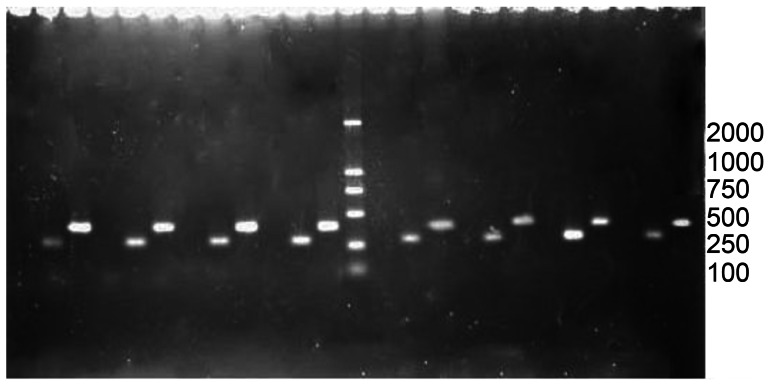
IL-1ra mRNA expression in the grafts. The level of IL-1ra mRNA gradually decreased with time. The marked 500 bp position corresponds to human 18s rDNA (internal control), and 280 bp corresponds to IL-1ra cDNA; the intrastromal injection group(group II) is to the right side of the marker, and the anterior chamber injection group(group III) is on the left; sample loading sequence in the two groups: from right to left: 3 days, before acute rejection, acute rejection, and two weeks after acute rejection.

RT-PCR was performed using total RNA extracted from the corneas in groups II and III as the template. The PCR products were analysed by agarose gel electrophoresis. Exogenous IL-1ra mRNA was expressed in the grafts, suggesting that hIL-1ra protein was expressed from the transcription, translation, and synthesis of hIL-1ra cDNA transfected into the cornea. In addition, at post-operative day 6, the mRNA expression in group II was higher than in group III (Fig 9).

## Discussion

Corneal graft rejection is a complex process. Evaluation of the mechanisms and treatment of graft rejection is important to improving the success of corneal transplantation. The Wistar-SD rat model used in this study is a high-risk corneal transplant model [11] in which donor rejection generally occurs 7 days after surgery, peaks between 10 and 14 days, and manifests with rapid corneal neovascularisation.

IL-1 is one of the key cytokines in corneal transplant rejection [13]–[14]. After stimulating corneal epithelial cells and corneal stromal cells with IL-1, studies have shown that the monocyte chemotactic factor secreted by corneal stromal cells increases 150 fold [4]. Niederkorn [15] demonstrated that Langerhans cells moved towards the centre of the cornea 30 minutes after injecting IL-1 into the cornea and mononuclear cells that infiltrated the cornea promoted antigen presentation and activated immune rejection.

Currently, the study of IL-1ra in the field of ophthalmology is in the early phase. Dana [8] reported that the topical application of 2% IL-1ra formulated in a hyaluronic acid vehicle blocked the activity of IL-1 in a mouse model of keratoplasty. The mean suppression of leukocyte infiltration after transplantation was 71%, and the rejection rate was 15%; therefore, topical IL-1Ra could promote graft survival. Although IL-1ra clearly inhibits immune and inflammatory reactions, the IL-1ra protein is not sufficiently stable for use in clinical applications, and developing an effective model for IL-1ra administration is clearly important research.

Previous studies on corneal gene therapy have typically transferred therapeutic genes into cells or grafts ex vivo before transplantation. Comer [16] used adenoviral vectors expressing CTLA-Ig (Ad CTLA) to transfect the corneas of Norway mice ex vivo and transplanted the transfected donor tissue into recipient Lewis mice, which prolonged the survival time of the corneal grafts. Klebe [17] cloned sheep IL-10 cDNA and transfected donor corneas with an adenoviral vector ex vivo, and they also observed similar protective effects on the grafts. Rayner [18] used a replication-defective virus as a vector to transfer a TNFR-Ig-encoding gene into rabbit corneas, and TNFR-Ig expression was detected within 4 weeks. However, corneal grafts transfected with empty vector showed severe inflammatory reactions, which may have accelerated corneal endothelial rejection [19]. These studies demonstrate the effectiveness of gene transfer in treating corneal rejection; however, the procedure for gene transfection ex vivo is highly complex and demands more extensive treatment conditions and longer transfection times. It is not practical to perform graft transfection for urgent cornea transplants. In addition, the safety of viral vectors for gene therapy in corneal graft rejection requires further improvement.

In our study, we used a cationic polymer as a vector for gene transfer. This polymer showed good biological compatibility and was able to reduce DNA degradation and prolong the expression of gene-coding sequences in target tissues. We injected the IL-1ra gene into donor corneas and anterior chambers during keratoplasty, and corneal rejection occurred later in the grafts that received the IL-1ra gene.

The analysis of the graft survival curves suggested that the corneal transparency rates in the IL-1ra gene-treated group and the IL-1ra protein-treated group were higher than that of the untreated group. The rate of rejection in the IL-1ra gene-treated group was less than that of the IL-1ra protein-treated group 12 days after the operation because IL-1ra protein maintained high local expression levels, which can inhibit the inflammatory reaction after transfecting corneal tissue through the IL-1ra gene in situ. By contrast, the effects of IL-1ra protein have a shorter duration because of its unstable properties, although it did reach a short-term high peak in the IL-1ra protein-injected group. However, IL-1ra protein expression was decreased, resulting in a diminished capacity to inhibit inflammation because of the gradual degradation of interior/exterior IL-1ra gene product in the corneal tissue. Therefore, the emphasis of future research should be to maintain high IL-1ra gene expression for an extended period after gene transfection.

Even after the rejection reaction, the corneal neovascularisation scores were lower in the gene treatment groups compared with the control group. Therefore, we believe that IL-1ra prolongs the time of graft transparency, not only by inhibiting IL-1 but also by inhibiting local neovascularisation. The results showed that a large number of CD4+ and CD8+ T cells and macrophages had infiltrated in the exterior of the cornea, and that RANTES and TGFβ1 expression was simultaneously up-regulated, particularly in the cells present during the acute rejection reaction phase. Activated T cells released a variety of lymphokines, which can induce the increased expression of MHC-II protein expression, ICAM-1 and other immune molecules in addition to macrophage infiltration, eventually causing delayed-type hypersensitivity and autoimmune damage to the corneal tissue. CD8+ cells present during the rejection of corneal tissue can kill donor-derived cells and produce specific cytotoxins that play a role in corneal graft rejection. These cellular and molecular changes were the likely basis of the immunopathological damage. to the graft. The observed cell infiltration and CD4+ and CD8+ T cell counts in the IL-1ra gene therapy groups were lower than in the control group, suggesting that IL-1ra can effectively inhibit the aggregation of immune effector cells in the graft.

TGF-β1 can induce anterior chamber-associated immune modulation and reduce corneal graft rejection [20]. The expression of TGF-β1 was positively correlated with the graft inflammatory response in our experiments, and TGF-β1 expression was higher in the control group than in the IL-1ra-treated groups. This finding indicates that corneal transplant rejection was not suppressed by TGF-β1 expression, suggesting a strong local immune response after corneal transplantation. Researchers have shown that TGF-β1 is one of the most powerful macrophage chemokines [21]. TGF-β1 promotes macrophage migration to the cornea, which can then act as antigen-presenting cells, secreting many cytokines, including IL-1, IL-6, IL-8 and tumour necrosis factor. These cytokines can induce a strong inflammatory response and may induce graft destruction. When expressed in the corneal graft, the IL-1ra gene produced IL-1ra protein and prevented the accumulation of macrophages in the grafts, thus weakening the negative impact of TGF-β1. The exact mechanisms of interaction should be further studied.

The binding of the inflammatory chemokine RANTES to its receptor can activate signal transduction pathways that regulate the biological activity of the target cells. In 1999, using a polymerisation RNA enzyme protection analysis system, Yamagami [22] detected RANTES gene expression in corneal tissue homogenates after transplanting mouse corneal allografts. They reported that the increased expression of mRNA for cytokines of the CYC (α) and CC (β) superfamilies of cytokines after allogeneic corneal grafts was related to corneal rejection. IL-1β promotes macrophage and T-cell tissue infiltration by inducing RANTES expression. T cells increase the expression of RANTES and attract more T cells and macrophages to sites of inflammation to amplify the reaction [23]–[24]. The results of our study confirm that RANTES is highly expressed in corneal grafts during early acute rejection and is maintained after rejection. RANTES expression in the IL-1ra treatment group was lower than in the control group. IL-1ra blockade of IL-1β prevented RANTES up-regulation in the corneal transplants, thus weakening the chemotaxis and activation of immune and inflammatory cells. The IL-1ra-induced down-regulation of the inflammatory response prolonged graft survival time.

## Conclusion

In this paper, we reported the efficacy of the treatment of corneal graft rejection with the IL-1ra gene by in situ corneal transfer mediated by a cationic polymer. High IL-1ra protein expression in the cornea down-regulated the expression of immune molecules and alleviated immune cell infiltration, which subsequently inhibited the immune rejection reaction and prolonged corneal graft transparency. As a new treatment model, IL-1ra gene therapy may help prevent and treat corneal graft rejection in humans.
